# Shorter Small-Sided Game Sets May Increase the Intensity of Internal and External Load Measures: A Study in Amateur Soccer Players

**DOI:** 10.3390/sports7050107

**Published:** 2019-05-09

**Authors:** Filipe Manuel Clemente, Pantelis Theodoros Nikolaidis, Thomas Rosemann, Beat Knechtle

**Affiliations:** 1 Polytechnic Institute of Viana do Castelo, School of Sport and Leisure, 4960-320 Melgaço, Portugal; 2 Exercise Physiology Laboratory, 18450 Nikaia, Greece; pademil@hotmail.com; 3 Institute of Primary Care, University of Zurich, 8091 Zurich, Switzerland; thomas.rosemann@usz.ch (T.R.); beat.knechtle@hispeed.ch (B.K.); 4 Medbase St. Gallen Am Vadianplatz, 9001 St. Gallen, Switzerland

**Keywords:** football, drill-based tasks, sided-games, intermittent exercises, training load, training monitoring

## Abstract

The purpose of this study was to compare internal and external load measures during two regimens (6 x 3’ and 3 x 6’) of a 5 vs. 5 format of play. Moreover, within-regimen changes (between sets) were also tested. Ten amateur soccer players (age: 19.8 ± 1.6 years; experience: 8.3 ± 2.1 years; height: 177.4 ± 3.8 cm; weight: 71.7 ± 4.2 kg) participated in the experiment. Internal load was measured using the CR-10 scale as the rated of perceived exertion (RPE) scale and a heart rate (HR) monitor. The measurements of total (TD), running (RD) and sprinting (SD) distances were also collected using a 10-Hz validated and reliable GPS. Comparisons between regimens revealed that the 3 x 6’ regimen was significantly more intense in terms of RPE than the 6 x 3’ regimen (p = 0.028; d = 0.351), although no significant differences were found in HR. Significantly greater averages of TD (p = 0.000; d = 0.871) and RD (p = 0.004; d = 0.491) were found in the 6 x 3’ regimen. In both regimens, the RPE was significantly lower during the first set than in the remaining sets. On the other hand, the TD was significantly shorter in the last sets than in the earlier. In summary, the present study suggests that shorter sets may be beneficial for maintaining higher internal and external load intensities during 5 vs. 5 formats, and that a drop-in performance may occur throughout the sets in both regimens.

## 1. Introduction

Small formats of play (typically called small-sided games (SSGs)) are often used in soccer training scenarios with the aims of maintaining the dynamic of the game and increasing the overall intensity of effort [1,2]. Usually, SSGs may vary in factors such as, among others, the number of players involved in the game (format), the size of the pitch, the type of scoring, the number of ball touches and the type of marking, compared to the real format of play, thereby aiming to fit the new version with the objectives of the session defined by the coach [3]. In this way, different versions of the game may emerge from the interaction of different task conditions and, if properly designed, the new games may contribute to a high and adjusted exertion level of the players [4], the development of tactical and technical skills and, possibly, physiological/physical capacities [5].

Typically, small-to-medium formats (2 vs. 2 to 6 vs. 6) promote heart rates (HRs) between 85 and 90% of the maximum and blood lactate concentrations between 4 and 8 mmol/l^−1^ [6]. Moreover, the distances covered may vary between 80 and 100 m/min^−1^ and the running distances between 6 and 10 m/min^−1^ [7]. The implications of different task conditions and their concurrency contribute to the changes in the acute responses in both internal and external load measures [8]. However, it is not only task conditions that may lead to different stimuli and implications. The type of exercise regimen is also an important factor contributing to changes in the overall load of the players [2,8]. 

Usually, intermittent regimens (i.e., interval training) are used in SSGs, mainly because of the highly demanding nature of these games. However, intermittent regimens can be planned in different ways, mainly involving the time of each set, the number of sets, the time of rest between them and the work:rest ratio. Considering that the manipulation of these elements may lead to different acute responses from the players, some studies have been conducted testing the consequences of different intermittent regimens of SSGs on performance [9,10]. Fanchini et al. [9] tested the 3 vs. 3 format in three regimens and found that long sets (3 x 6 min/2 min rest) contributed to a drop in heart rate responses compared to medium (3 x 4 min/2 min rest) and short (3 x 2 min/2 min rest) sets, although no significant difference was observed for perceived effort or technical actions [9]. Also, comparing the internal and external load variations between training regimens, it was observed that shorter sets elicited a lower maximal heart rate, a shorter distance covered at low running speed, and contributed to an increase in the distances covered at both medium and high running speeds [10]. Despite this evidence, the number of studies comparing different intermittent regimens in SSGs is not enough to clarify the real impact on the players and to generalize the information.

It is expected that a good adjustment of SSGs and associated training regimens may increase the chances to optimize the training process and increase the beneficial effects on the players’ performance. Nevertheless, the low number of studies about different intermittent regimens of SSGs and the consequences for the load [9,10] do not provide clear evidence that may help coaches to understand the real consequences of different regimens in terms of the overall stimulus per set and the possible drop in performance throughout the exercise. For this reason, a comparison between set durations may help to choose the most appropriate regimens to fit with the objectives of the training plan. Considering the above-mentioned reasons, the purpose of this study was to analyze the variations of internal and external load measures between two types of 5 vs. 5 format regimen, and also to compare the variations of such measures within the regimen (between sets). 

## 2. Materials and Methods

### 2.1. Ethical Approval

The present study design was analyzed and approved by a local ethical committee (Polytechnic Institute of Viana do Castelo, School of Sport and Leisure) with the following identification number: IPVC-ESDL180602. The study was conducted based on the ethical standards for the study of humans as recommended by the Declaration of Helsinki.

### 2.2. Participants

Ten male amateur soccer players (age: 19.8 ± 1.6 years; experience: 8.3 ± 2.1 years; height: 177.4 ± 3.8 cm; weight: 71.7 ± 4.2 kg) participated in this study. These players belong to the same team and usually train four times a week plus one match every weekend. Before the study began, the experimental procedures were explained in detail to the participants. The potential risks and benefits were also explained. After the explanation, the participants voluntarily signed an informed consent document. 

### 2.3. Study Design

A cross-sectional study design was conducted to analyze the variations in rating of perceived exertion (RPE), mean heart rate (HR mean), total distance (TD), running distance (RD) and sprinting distance (SD) between two 5 vs. 5 regimens (6 sets of 3 minutes with 2 minutes of rest [6 x 3’/2’ regimen] and 3 sets of 6 minutes with 2 minutes of rest [3 x 6’/2’ regimen]). The experiments were carried out on four days split across the last two weeks of the season (2 days of data collection per week). On the first day, the 6 x 3’/2’ regimen was carried out followed by the 3 x 6’/2’ regimen after two days. On the second week, the opposite was performed (3 x 6’/2’ on the third day and 6 x 3’/2’ on the fourth day of data collection). Before each session began, the players were informed about the regimen to be played so that they could be prepared for the load management. A 72-hour period interspaced the second from the third day of data collection.

With the aim of homogenizing the teams, the ten players were previously classified based on their skill performance using the Team Sport Assessment Procedure (TSAP) [11]. To measure this, the players participated in a 5 vs. 5 format of play for 2 games of 10 min each in the week before the study began. The games were recorded using a video camera and assessed using the TSAP instrument to measure the performance score of each player. Using the performance score of each player and combining such information with the playing position, two teams with similar performance score levels and playing positions were constituted. The teams remained constant during the data collection sessions. The observations were made by two expert observers who had an inter-reliability level of 0.93 (intra-class correlation, ICC) and intra-reliability level of 0.97 (ICC), measured in a previous pre–post pilot study with a 20-day interval using the same observational instrument. 

The external load of the players was monitored using a 10-Hz GPS (Johan Sports company, Hoofddorp, The Netherlands) and the internal load using a HR monitor (H7, Polar, Finland), during the 5 vs. 5 games. All the games were played on synthetic turf between 17:00 and 18:00 at an average temperature of 21.1 ± 1.3 °C and a relative humidity of 63.4 ± 2.6%. A standardized warm-up protocol was employed before each session of data collection. The warm-up consisted of 5 minutes of low-to-moderate running (self-paced), followed by lower-limb dynamic stretching and mobility exercises. Agility and speed drills were also conducted, and 5 min of a ball possession game within a space of 20 x 20 m was also performed. 

### 2.4. The 5 vs. 5 Game

A 5 vs. 5 format with small goals (2 x 1 m) and no goalkeepers was implemented in a 30 x 30 m pitch (90 m^2^/player). The format was executed with two training regimens: 6 x 3’/2’ rest and 3 x 6’/2’ rest. 

No verbal feedback was given to the players before, during or after the games. Nevertheless, verbal encouragement (with no tactical or technical content) was given during the games with the aim of maximizing and maintaining levels of commitment and effort. Official soccer rules were kept with exceptions of (1) offside (not considered), (2) means of scoring (in the small-goals), and (3) ball replacements (with the foot). 

### 2.5. External Load

A 10-Hz validated and reliable GPS with an accelerometer, gyroscope, and magnetometer (100 Hz, 3 axes) (Johan Sports company, Hoofddorp, The Netherlands) [12] was used by each player during the games. The following measurements were collected in each set: (a) total distance (TD); (b) running distance (14–19.9 km/h^−1^) (RD); and (c) sprinting distance (> 20.0 km/h^−1^) (SD). The data was standardized per min to execute the comparisons.

### 2.6. Internal Load (RPE)

The internal load was determined using a subjective questionnaire and an objectively measured instrument. Borg’s CR-10 scale [13] was employed immediately after each set. In that scale, 1 represents a very, very light activity and 10 represents the maximal exertion. The players performed their ratings individually so that they could not hear or be influenced by the answers of the other players. Aiming to reduce the intra-variability of the answers, the players were familiarized with the scale in the weeks prior to the beginning of the study.

In order to objectively measure the internal load, each player used an HR monitor (H7, Polar, Finland) with a data sample rate of 1 s. The data was uploaded and processed on Polar software (Polar, Finland). The average HR per player was collected during all the games. 

### 2.7. Statistical Procedures

The average of the sets of two sessions for each SSG regimen was used to make the comparisons. Preliminary tests did not find significant inter-session variations for each SSG regimen. The variations in internal and external load measures between 5 vs. 5 regimens were tested using a mixed-design ANOVA followed by a post hoc Bonferroni test, after confirmation of the normality and homogeneity assumptions. The tests were executed using SPSS software (version 24.0, IBM, USA) for a p < 0.05. The effect size (ES) comparisons were executed using the Cohen d (d) and the inferences of magnitude were made with the following thresholds [14]: [0.0;0.2], trivial; [0.2;0.6], small; [0.6;1.2], moderate; [1.2;2.0], large; >2.0, very large. 

## 3. Results

**Figure 1:**
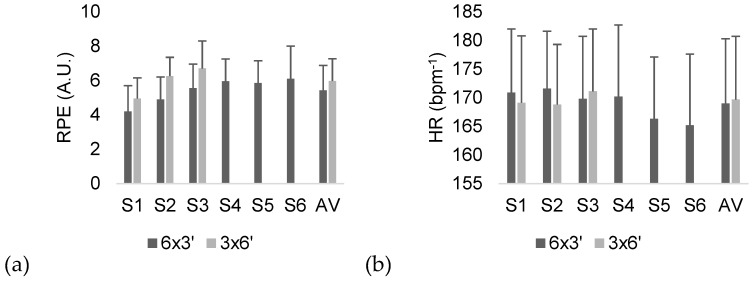
Descriptive statistics (average and SD) of the (**a**) RPE (rating of perceived exertion) and (**b**) HR (heart rate) of the players during the two regimens. S: set; AV: average of the sets.

The descriptive statistics of RPE and HR can be found in Figure 1. The comparisons of external load and internal load variables between SSG regimens can be found in Table 1.

The average of all sets reveals that the 3 x 6’ regimen was significantly more intense in terms of RPE than the 6 x 3’ regimen (difference of averages [dif]: 0.54 arbitrary units (A.U.); p = 0.028; d = 0.351, small effect). Considering HR, no significant variations between the regimens (dif: 0.65 bpm^−1^; p = 0.716; d = 0.058, trivial effect) were found. 

**Figure 2:**
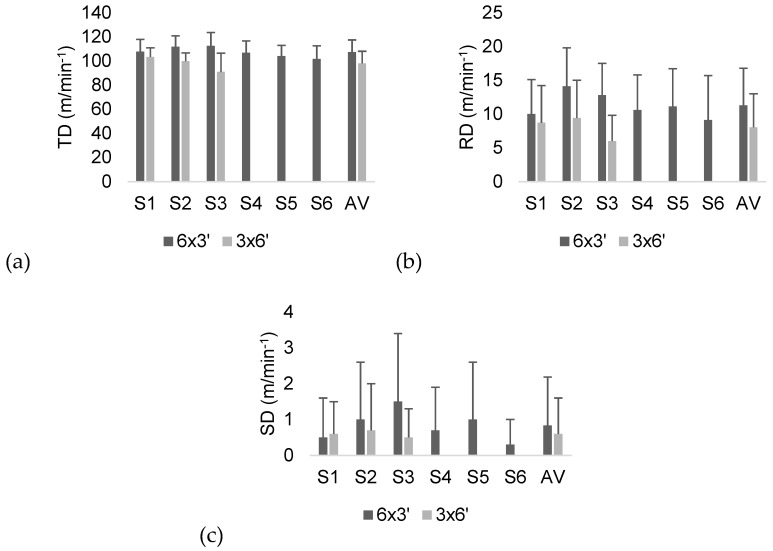
Descriptive statistics (average and SD) of the (**a**) TD (total distance), (**b**) RD (running distance) and (**c**) SD (sprinting distance) of the players during the two regimens. S: set; AV: average of the sets.

In Figure 2, we present the descriptive statistics of external load measures during the games. Significantly greater TD averages were found in the 6 x 3’ regimen (dif: 9.53 m/min^−1^; p = 0.000; d = 0.871, moderate effect). Considering the RD, significantly greater distances were covered in the 6 x 3’ regimen (dif: 2.41 m/min^−1^; p = 0.004; d = 0.491, small effect). No meaningful differences in SD were found between regimens (dif: 0.87 m/min^−1^; p = 1.43; d = 0.097, trivial effect).

Within-regimen comparisons were performed to analyze the variations in external and internal load measures between the sets. Considering the RPE during the 6 x 3’ regimen, the first set was significantly lower than the fourth (dif: 1.75 A.U.; p = 0.004; d = 1.25, large effect), fifth (dif: 1.65 A.U.; p = 0.008; d = 1.185, moderate effect) and sixth (dif: 1.90 A.U.; p = 0.001; d = 1.12, moderate effect) sets. Moreover, the players covered a significantly shorter TD during the sixth set of 6 x 3’ than in the second (dif: 10.16 m/min^−1^; p = 0.34; d = 1.018, moderate effect) and the third (dif: 10.95 m/min^−1^; p = 10.95; d = 0.996) sets. No significant within-6 x 3’ regimen changes were found in the HR, RD and SD (p > 0.05).

Variations in RPE between sets in the 3 x 6’ regimen revealed significantly lower rate averages in the first set than in the second (dif: 1.30 A.U.; p = 0.007; d = 1.149, moderate effect) and the third sets (dif: 1.75 A.U.; p = 0.000; d = 1.261, large effect). Considering the TD, a significantly shorter TD was covered in the third set compared to the first set (dif: 12.39 m/min^−1^; p = 0.005; d = 1.009, moderate effect). Finally, in the same regimen (3 x 6’), a significantly shorter RD was covered during the third set than in the second (dif: 3.97 m/min^−1^; p = 0.014; d = 1.091, moderate effect). No significant within-6 x 3’ regimen changes were found in the HR and SD (p > 0.05).

## 4. Discussion

The present study revealed that the shorter sets resulted in significantly increased RPE intensities and total and running distances covered by the players. Moreover, it was also found that in both training regimens the lowest RPE intensities occurred in the first set and, on the other hand, the shorter TDs in the last set.

No significant changes were found between and within regimens. One possibility to explain the absence of differences between HR was that the shorter regimen had a duration of 3 min witch involves a sufficient level of aerobic stimulus. The absence of difference is in line with previous studies comparing heart rate responses between intermittent regimens [9,15]. Nevertheless, the perceived measure of intensity (RPE) revealed significant differences between the regimens, namely lower rates in the shorter sets (6 x 3’). This may be related to the psychobiological effects of time of exertion; thus, coaches should be aware of such consequences considering the potential acute physiological fatigue effect. Besides the differences between formats in terms of RPE, it was also possible to identify an increasing tendency to report higher efforts throughout the sets in both training regimens. However, the multidimensional fatigue effect may be the cause of such RPE increases, especially considering that decreases in total and running distances and player load occurred across the sets in both regimens [16].

The consequences of the number of sets on external load variables were also found, namely in the TD and RD. In the case of 6 x 3’, a progressive decrease in TD was observed. The sixth and last set presented significantly lower TDs than the second and the third sets. This result suggests that the drop in TD occurs in the last sets and this may be due to the continuity of exertion [17]. Similar evidence was observed in the 6 x 3’ regimen. However, in this one, progressive and significant decreases across the sets were also found in the RD, which is an important variable that may contribute to a proper moderate-to-high aerobic stimulus. For that reason, shorter sets seem not to contribute to a significant decrease in RD and this should be considered by coaches when choosing a regimen for RD stimulation. Differences in SD were not found, possibly because of the very small and highly variable number of actions performed at this speed [7] during a 5 vs. 5 format game. 

The overall shorter TD and RD during longer sets may be due to the players’ awareness of the time of each game and the consequences on self-organization of pace. In a previous study about this possibility, different intermittent regimens and their effects on pacing were compared, revealing that high-speed distances progressively and significantly decreased across shorter sets (i.e., 1 min) based on the ‘all-out’ pacing strategy used by rugby players [17]. Conversely, during longer sets, a more constant high-speed pace across the sets was observed [17]. The results of the present study are in line with these previous findings. However, this constancy in the most intense activities occurring in longer sets also resulted in lower values when compared with shorter sets.

It is important to highlight some limitations of our study. The small number of participants and also their amateur level may be considered as limitations that should be considered in future studies. Moreover, the fitness levels were not assessed, and this can be a determinant factor in sustaining higher levels across sets. More formats should be analyzed in future studies, considering that the load varies accordingly to the format of play. Finally, different work-to-rest ratios should be considered in future study designs in order to analyze the effects of rest on physical demands and the internal effect of exercise.

Despite the above-mentioned limitations, the results presented in our study suggest that shorter sets (3’) seem to be more beneficial than longer sets (6’) in maintaining total and running distances while decreasing the RPE. Additionally, the fatigue effect seems to mostly occur in later sets. Coaches may use such information to choose shorter periods of exertion with a greater number of sets (bouts) to maintain a high level of physical demand and to contribute to an optimization of the high-energy systems that support highly demanding actions.

## 5. Conclusions

This study revealed that shorter sets contribute to a significant increase in the TD and RD of players and maintain lower rates of perceived effort, in comparison to longer sets. Moreover, the earlier sets present lower perceived effort rates and greater TD and RD averags.

## Figures and Tables

**Table 1:** Differences in the averages (6 x 3’-3 x 6’) of external and internal load variables between SSG regimens.

	Dif (6 x 3’)-(3 x 6’)	p	d	Inference of ES
RPE (A.U.)	−0.54	0.028	0.351	Small
HR (bpm^−1^)	−0.65	0.716	0.058	Trivial
TD (m/min^−1^)	9.53	0.000	0.871	Moderate
RD (m/min^−1^)	2.41	0.004	0.491	Small
SD (m/min^−1^)	0.13	0.568	0.097	Trivial

RPE: (rating of perceived exertion); HR: heart rate; TD: total distance; RD: running distance (14–19.9 km/h^−1^); SD: sprinting distance (>20.0 km/h^−1^); Dif: differences in the averages between 6 x 3’ and 3 x 6’ regimens; p = p-value; d = Cohen d; ES: effect size.
